# Prediction models for post-stroke delirium: a systematic review with an exploratory meta-analysis of predictors

**DOI:** 10.3389/fneur.2026.1800718

**Published:** 2026-06-18

**Authors:** Weiya Ma, Sumin Ma, Qiaomin Tang, Yuanyuan Sun, Chen Hu

**Affiliations:** Department of Nursing, The Second Affiliated Hospital of Zhejiang University School of Medicine, Hangzhou, China

**Keywords:** delirium, meta-analysis, prediction model, stroke, systematic review

## Abstract

**Objective:**

To systematically identify and synthesize predictors of post-stroke delirium (PSD) derived from existing prediction models, and to assess the methodological quality of these studies using PROBAST.

**Methods:**

A comprehensive systematic search was conducted in nine databases from inception to April 2026. Studies developing or validating prediction models for PSD were included. Data extraction was guided by the CHARMS checklist. Methodological quality and risk of bias were assessed using the Prediction Model Risk of Bias Assessment Tool (PROBAST). Meta-analysis was performed to pool the effect sizes of predictors and the area under the receiver operating characteristic curve (AUC).

**Results:**

Sixteen studies (24 models) with sample sizes ranging from 100 to 14,475 were included. Model discrimination was moderate to good, with reported AUC values ranged from 0.72 to 0.94. The meta-analytic pooled AUC was 0.83 (95% Confidence interval: 0.81–0.85). Age, NIHSS (National Institutes of Health Stroke Scale score), neutrophil-to-lymphocyte ratio, visual impairment, and infection were identified as common significant predictors. PROBAST assessment revealed a high overall risk of bias in all studies, primarily due to methodological shortcomings in the analysis domain. Calibration was assessed in six studies with acceptable performance, whereas clinical utility was rarely evaluated.

**Conclusion:**

This study highlights several important predictors of PSD. However, due to the high risk of bias, the reliability of existing models remains uncertain. Although the pooled AUC of 0.84 suggests moderate to good discrimination, its performance in individual clinical settings may vary markedly. Future studies should adhere to unified PSD diagnosis criteria, employ robust validation strategies, and explore advanced modeling techniques to improve model reliability and clinical utility.

## 1 Introduction

Stroke remains one of the leading causes of mortality and long-term disability worldwide, imposing a substantial burden on healthcare systems (1). Post-stroke delirium (PSD), a common and serious neuropsychiatric complication, is characterized by an acute and fluctuating disturbance in attention, awareness, and cognition (2). PSD has been reported to occur in approximately 9.6 to 66.6%, with incidence varying widely depending on patient characteristics and assessment methods (3). The occurrence of PSD is associated with a range of adverse clinical outcomes, including prolonged hospitalization, increased healthcare costs, functional decline, cognitive impairment, institutionalization, and higher mortality rates (4, 5). Importantly, delirium is often underdiagnosed in clinical practice, particularly in non-intensive care settings, due to its fluctuating nature and overlapping symptoms with other neurological deficits following stroke (6). Early identification of patients at high risk for PSD is considerable of clinical importance. However, there is currently no widely accepted or implemented prediction tool for PSD in clinical practice. Timely risk stratification may facilitate targeted preventive interventions, optimized monitoring, and individualized management strategies.

In recent years, numerous multivariable prediction models have been developed to estimate the risk of delirium following stroke. These models incorporate a variety of demographic, clinical, laboratory, and imaging variables, and are intended to support clinical decision-making. Despite the growing number of proposed models, their methodological rigor, predictive performance, and clinical usefulness remain uncertain. To date, few systematic reviews have critically evaluated PSD prediction models using established methodological appraisal tools.

Therefore, this study aimed to systematically identify predictors of PSD derived from existing prediction models, synthesize their effect sizes, and evaluate the methodological quality of the included studies using PROBAST.

## 2 Method

This study was conducted under the Preferred Reporting Items for Systematic Reviews and Meta-Analyses (PRISMA 2020) guidelines (7).

### 2.1 Search strategy

A systematic literature search was conducted in the following databases: Web of Science, the Cochrane Library, PubMed, Embase, CINAHL, China National Knowledge Infrastructure (CNKI), Wanfang Data Knowledge Service Platform, Chinese Medical Journal Full-text Database, and the Chinese Biomedical Literature Database (CBM). Both controlled vocabulary (e.g., MeSH terms) and free-text terms were used in combination. In addition, we also conducted supplementary searches by reviewing the reference lists of the included articles. The detailed search strategy can be found in Supplementary Table S1. This systematic review was conducted according to the PICOTS framework. The details are as follows:

- P (population): adult patients with ischemic or hemorrhagic stroke.
- I (index): models for predicting the risk of PSD (including ≥2 predictors).
- C (comparison): not applicable.
- O (outcome): prediction of the occurrence of PSD.
- T (timing): demographic characteristics, clinical assessment scale scores, and laboratory indicators were collected at hospital admission or during the acute phase of stroke and were used to predict the subsequent occurrence of post-stroke delirium during hospitalization or within a defined follow-up period.
- S (setting): in hospital-based clinical settings.

### 2.2 Eligibility criteria

#### 2.2.1 Inclusion criteria

(1) Adult patients (≥18 years) with stroke; (2) Developed, validated, or updated prediction models for estimating the risk of post-stroke delirium; (3) Employed an observational study design, including cohort study, case–control study, or cross-sectional study); (4) Published in Chinese or English.

#### 2.2.2 Exclusion criteria

(1) Studies that reported only risk factors without developing or validating prediction models; (2) studies with insufficient information for data extraction; (3) studies for which the full text was unavailable; and (4) dissertations and conference abstracts.

### 2.3 Study screening and data extraction

Duplicates were removed using Zotero software. Two reviewers independently screened the literature and extracted data according to the predefined inclusion and exclusion criteria, with cross-checking performed to ensure consistency. Any discrepancies were resolved through discussion or adjudication by a third reviewer. An Excel-based data extraction form was developed in accordance with the CHARMS checklist for systematic reviews of prediction model studies (8). The extracted information included the first author, year of publication, country, study design, study population, delirium assessment tools, sample size, number of delirium events, candidate predictors, model development and validation methods, and model performance measures.

### 2.4 Quality assessment

Methodological quality of the included studies was independently assessed by two reviewers using the Prediction Model Risk of Bias Assessment Tool (PROBAST) (9). This tool evaluates risk of bias across four domains: participants, predictors, outcome, and analysis, comprising a total of 20 signaling questions. Each signaling question was answered as “yes/probably yes,” “no/probably no,” or “no information.” A study was judged as having an overall low risk of bias if all four domains were rated as low risk. A study was judged as having an overall high risk of bias if at least one domain was rated as high risk. If at least one domain was rated as unclear risk while all remaining domains were rated as low risk, the overall risk of bias was judged as unclear.

### 2.5 Statistical analysis

Meta-analyses of predictors included in the prediction models were performed using Review Manager (RevMan) software (version 5.4), and results were reported as odds ratios (ORs) with corresponding 95% CI. The extracted ORs were derived from multivariable models. R software (version 4.5.2) was used to pool the AUC of eligible models and to generate forest plots. We prioritized pooled AUCs derived from validation datasets whenever available. To ensure comparability across studies, only studies employing standardized and widely validated diagnostic criteria for delirium (i.e., CAM, CAM-ICU, or DSM-V) as the reference standard were included to the meta-analysis of AUCs values. Studies using non-standardized or heterogeneous diagnostic frameworks, as well as those applying multiple delirium assessment tools, were excluded from the meta-analysis of AUCs values. For meta-analysis, AUCs were transformed using the logit transformation to stabilize variance. Prediction intervals were calculated based on a random-effects model to estimate the expected range of AUC values in future studies, incorporating both within-study uncertainty and between-study heterogeneity. The pooling of AUC was considered exploratory and interpreted with caution. Statistical heterogeneity among included studies was assessed using the *I*^2^ statistic and Cochran’s *Q* test. A fixed-effects model was applied when no significant heterogeneity was detected (*p* > 0.10 and *I*^2^ < 50%). Otherwise, a random-effects model was used when significant heterogeneity was present (*p* ≤ 0.10 and *I*^2^ ≥ 50%).

## 3 Results

### 3.1 Study selection and characteristics

**Figure 1:**
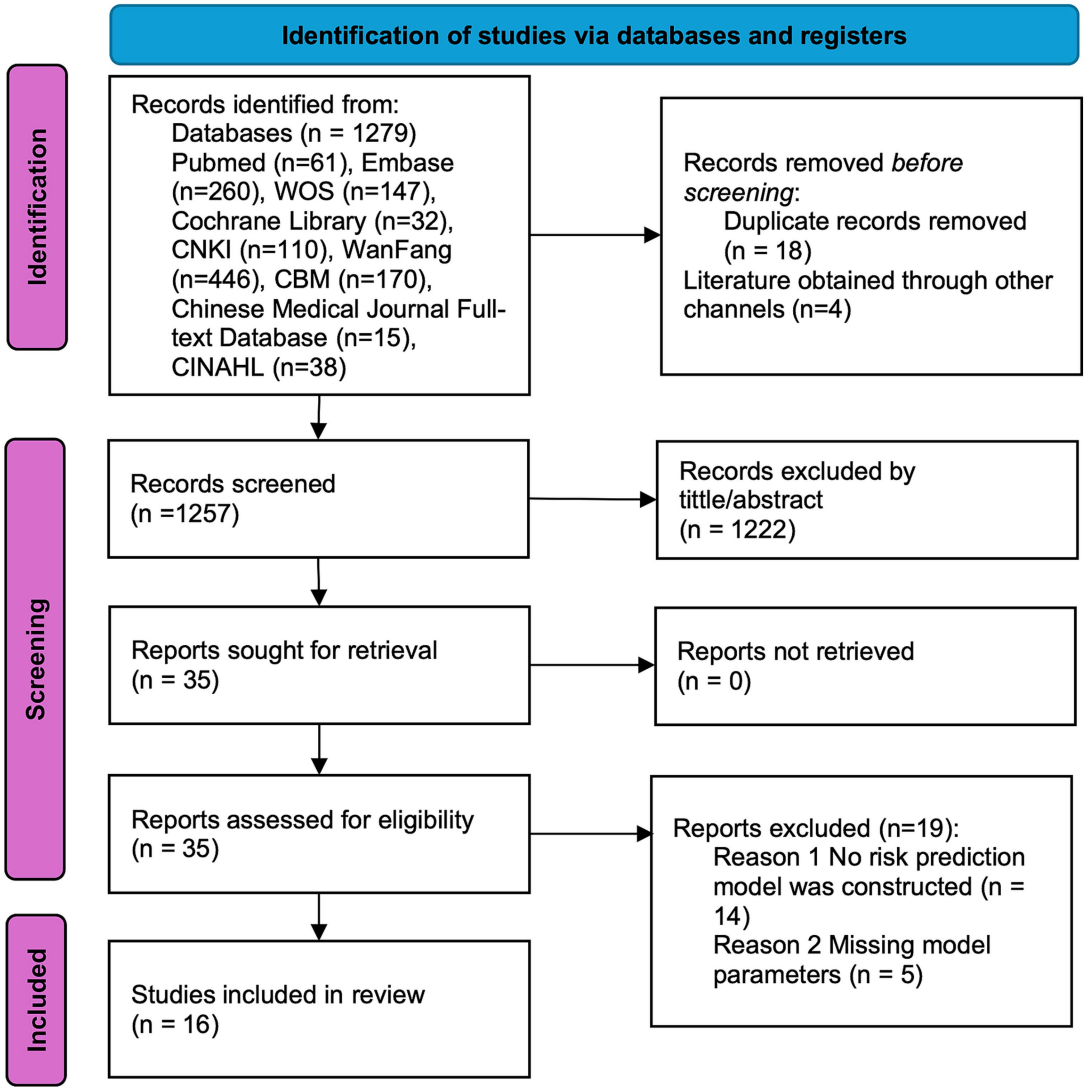
Preferred reporting items for systematic reviews and meta-analyses diagram.

The literature search initially identified 1,279 records. After screening, 16 studies were included in the systematic review and meta-analysis. The study selection process is presented in Figure 1.

### 3.2 Study characteristics

The characteristics of the included studies are summarized in Table 1. All 16 included studies adopted observational study designs, encompassing retrospective and prospective cohort studies. Sample sizes ranged from 100 to 14,475 participants. The reported incidence of post-stroke delirium varied widely, ranging from approximately 2.7 to 43%.

**Table 1:** Summary of included studies on prediction models for PSD.

Author	Year	Country	Stroke type	PSD cases/sample size	Training group sample size	Study design	Delirium diagnostic criteria	Candidate variables (*n*)
Berger et al. (19)	2025	Austria	Acute ischemic stroke	398/14475 (2.7%)	6,151	Retrospective cohort study	ICD-10 diagnostic codes	50
Cai et al. (15)	2025	China	Ischemic stroke	105/497 (21.1%)	347	Prospective observational study	3D-CAM	32
Cui et al. (16)	2025	China	Stroke	141/502 (28.1%)	502	Prospective observational study	CAM-ICU	19
Guldolf et al. (10)	2021	Belgium	Acute ischemic stroke	201/514 (39.1%)	514	Retrospective cohort study	DSM-V	17
Haight and Marsh (22)	2020	UnitedStates	Ischemic stroke or intracranial hemorrhage	51/202 (25.2%)	102	Retrospective cohort study	CAM-ICU	14
Kostalova et al. (20)	2012	Czech Republic	Cerebral infarction or intracerebral hemorrhage	43/100 (43%)	100	Prospective observational study	DSM-IV	37
Oldenbeuving et al. (13)	2014	Netherlands	Stroke	79/527 (15.0%)	527	Prospective observational study	CAM	7
Pasinska et al. (23)	2018	Poland	Stroke	203/750 (38.5%)	750	Prospective observational study	DSM-V	35
Zhou et al. (17)	2024	China	Acute ischemic stroke	158/572 (27.6%)	400	Retrospective cohort study	CAM	25
Wang et al. (24)	2025	China	Subarachnoid hemorrhage	42/252 (16.7%)	252	Prospective observational study	CAM-ICU	18
Fan et al. (21)	2022	China	Acute ischemic stroke	107/500 (21.4%)	350	Prospective observational study	CAM-ICU, 4AT	22
Nakamizo et al. (11)	2020	Japan	Acute stroke	42/387 (10.85%)	387	Prospective cohort study	Intensive care delirium screening checklist	20
Klimiec-Moskal et al. (14)	2023	Poland	Acute stroke	134/459 (29.19%)	459	Retrospective cohort study	CAM-ICU	12
Wischmann et al. (18)	2023	Germany	Acute stroke	98/445 (22.02%)	345	Retrospective cohort study	DSM-V	51
Rex et al. (12)	2024	United States	Hemorrhagic stroke patients	58/139 (41.73%)	139	Retrospective cohort study	DSM-V	9
Kotfis et al. (25)	2019	Poland	Ischemic stroke patients	172/1001 (17.18%)	1,001	Prospective cohort study	DSM-V	22

3D-CAM, 3-minute diagnostic interview for CAM; CAM-ICU, confusion assessment method for the intensive care unit; DSM, diagnostic and statistical manual of mental disorders; CAM, confusion assessment method; 4AT, the 4 “A”s test.

### 3.3 Model development and predictors

A total of 24 prediction models were constructed in the 16 included studies. Three studies developed two models each (10–12), one study developed three models (13), and one study developed four models (14). Logistic regression was the predominant modeling approach employed across the included studies. The final prediction models incorporated between three and eight predictor variables. Frequently reported predictors included demographic variables (e.g., age, sex), stroke severity indices (e.g., National Institutes of Health Stroke Scale [NIHSS] score), comorbidities, laboratory parameters, and functional or cognitive assessment scores. Several predictors were consistently identified across multiple models, including advanced age, higher NIHSS scores, infection, visual impairment, and elevated Neutrophil-to-Lymphocyte Ratio (NLR). Detailed information on candidate predictors and model specifications is provided in Table 2.

**Table 2:** Characteristics of included prediction model studies (*n* = 16).

Included studies	Model number	Main modeling method	Internal validation	External validation	Calibration method	Development AUC	Validation AUC	Model presentation	Predictors
Berger et al. (2025) (19)	1	Multivariable logistic regression analysis	Random split	—	Calibration curve: good agreement	0.72 (0.69–0.75)	0.72 (0.70–0.74)	Scoring system	8 predictors: previous delirium, chronic alcohol consumption, age >70 years, male sex, infection, NIHSS score, non-lacunar stroke, visual/hearing impairment
Cai et al. (2025) (15)	1	Univariate analysis, multivariate logistic regression analysis	Random split	—	Calibration curve: good fit; H-L test (*p >* 0.05)	0.885	0.865	Nomogram	4 predictors: coronary heart disease, indwelling urinary catheter, physical restraint, NLR
Cui et al. (2025) (16)	1	Univariate analysis, multivariate logistic regression analysis	1,000 bootstrap	—	Calibration curve: good fit	0.92	0.90	Nomogram	5 predictors: age, visual impairment, post-stroke infection, NIHSS, use of restraints
Guldolf et al. (2021) (10)	2	Multivariable logistic regression analysis	—	—	—	0.84 (0.81–0.88)	—	Table	5 predictors: NLR, age, NIHSS, modified Rankin Scale (mRS), cognitive impairment
						0.82 (0.78–0.86)			
Haight and Marsh (2020) (22)	1	Multivariable logistic regression analysis	—	Temporal external validation	—	0.90	0.82	Formula: log odds of delirium = 3.621 + (1.370) * (Age > 64 years) + (2.784) * (cognitive deficit/aphasia/neglect) + (1.842) * (AKI) + (1.350) * (intubation) + (3.619) * (presence of IVH)	6 predictors: age >64 years, intraventricular hemorrhage, intubation, cognitive impairment, aphasia or neglect, acute kidney injury
Kostalova et al. (2012) (20)	1	Multivariable logistic regression analysis	2-fold cross-validation	—	—	—	—	Formula: Log odds of delirium = −8.10 + 0.08 × Age + 1.58 × (GGT > 1.02) + 1.31 × ([bilirubin > 20]) + 1.81 × (ICH) + 1.38 × (Lesion > 40 cm^3^)	4 predictors: age, intracerebral hemorrhage, lesion volume >40 cm^3^, elevated *γ*-glutamyl transpeptidase
Oldenbeuving et al. (2011) (13)	3	Logistic regression analysis	—	Single-center prospective external validation	—	0.85 (0.80–0.90)	0.90	Scoring system	4 predictors: age, NIHSS, stroke subtype, infection
						0.84 (0.80–0.89)			
						0.80 (0.75–0.85)			
Pasinska et al. (2019) (23)	1	Univariate analysis, Multivariate logistic regression analysis	—	—	—	—	—	Table	6 predictors: admission MoCA score, white blood cell count, neglect, visual deficit, physical disability, pre-stroke comorbidities
Zhou et al. (2024) (17)	1	Univariate analysis, Multivariate logistic regression analysis	—	Temporal external validation	Calibration curve: good agreement	0.797 (0.745–0.851)	0.844 (0.781–0.908)	Nomogram	5 predictors: age, cerebrovascular intervention, high-sensitivity C-reactive protein, smoking, NIHSS
Wang et al. (2025) (24)	1	Multivariate logistic regression analysis	—	—	H-L test (*p* = 0.733)	0.890 (0.829–0.951)	—	Table	6 predictors: history of alcohol use, coronary heart disease, mechanical ventilation, analgesic use, sedative use, hypoalbuminemia
Fan et al. (2022) (21)	1	Multivariate logistic regression analysis	Temporal Internal Validation		H-L test (*p* = 0.183)	0.935 (0.897–0.973)	—	Formula: Z = 0. 036 × Age + 0. 475 × NIHSS + 0. 127 × NLR + 1.710 × Atrial fibrillation − 10.160	4 predictors: age, NIHSS, NLR, atrial fibrillation
Nakamizo et al. (2020) (11)	2	Univariate analysis, Multivariate logistic regression analysis	4,000 bootstrapping	—	—	0.78 (0.71–0.86) 0.81 (0.72–0.89)	—	Scoring system	5 predictors: prior delirium, alcohol, NIHSS ≥ 5, dementia, auditory/visual impairment
Klimiec-Moskal et al. (2023) (14)	4	Univariate analysis, Multivariate logistic regression analysis	—	—	H-L test (*p* = 0.532 for model A and *p* = 0.253 for model B)	0.77 (0.81–0.88)	—	Table	6 predictors: NIHSS score on admission, Atrial fibrillation, Diabetes mellitus, Pre-stroke dependency, Hemorrhagic stroke, CRP > 7.09 mg/L
						0.81 (0.77–0.85)			
						0.80 (0.76–0.84)			
						0.84 (0.80–0.88)			
Wischmann et al. (2023) (18)	1	5-fold cross-validation recursive feature elimination, Logistic regression analysis	5-fold cross-validation	Temporal external validation	—	0.8860	0.8498	Scoring system	6 predictors: GCS < 15 points at admission, Fazekas Score≥1 point in non-contrast computed tomography, Global brain atrophy in non-contrast computed tomography, Age≤68 years, NIHSS ≤1 point, Premodified Rankin Scale = 0 points
Rex et al. (2024) (12)	2	Univariate analysis, Multivariate logistic regression analysis	—	—	—	0.78 (0.71–0.86)	0.81 (0.72–0.89)	Scoring system	3 predictors: white matter hyperintensity volume, GCS > 13, small intracerebral hemorrhage
Kotfits et al. (2019) (25)	1	Univariate analysis	—	—	—	0.801 (0.7763–0.8257)	—	Formula: DELIAS score = 1.272 × Hemianopia + 0.098 × Dysphasia + 0.026 × Age + 0.054 × NIHSS − 0.005 × NLR + 0.028 × WBC	6 predictors: Leucocyte count, Neutrophil count, NLR (mean), NLR > 4.86 CRP, CRP > 9.10

AUC, area under the curve; ICH, intracerebral hemorrhage; H-L test, Hosmer–Lemeshow test; OR (Odds), odds ratio; AKI, acute kidney injury; GGT, gamma-glutamyl transferase; NLR, neutrophil-to-lymphocyte ratio; NIHSS, National Institutes of Health Stroke Scale; GCS, Glasgow Coma Scale; CRP, C-reactive protein; WBC, white blood cell count. “—” indicates not reported.

### 3.4 Model performance

Among the 16 included studies, 14 evaluated model discrimination using the AUC, with reported values ranging from 0.720 to 0.935, indicating moderate to good discriminatory performance. Among these, three studies reported AUC values exceeding 0.850 in the validation datasets (13, 15, 16). Calibration performance was assessed in seven studies using calibration plots or the H-L goodness-of-fit test. Overall, the included models demonstrated acceptable calibration performance. However, only one study assessed clinical utility using decision curve analysis (16). This limited evaluation of net benefit suggests that the clinical applicability and practical value of existing PSD prediction models remain insufficiently explored. Three models presented risk prediction in the form of nomograms (15–17). Three nomograms demonstrate favorable feasibility for bedside application, with no complex calculation tools required, short assessment time, and good user-friendliness, which is consistent with other findings (11, 18). They also show high potential for integration into electronic health records, as all predictor variables are routinely available in structured electronic health record data.

### 3.5 Model validation

Among the included studies, seven performed internal validation (11, 15, 16, 18–21), four conducted external validation (13, 17, 18, 22). One carried out both internal and external validation (18). The remaining six studies did not perform either internal or external validation (10, 12, 14, 23–25). Internal validation was performed using k-fold cross-validation in two studies (18, 20), a random split-sample approach in two studies (15, 19), a temporal internal validation in one study (21), and bootstrap resampling techniques in another two studies (11, 16). Three studies performed temporal external validation within the same sizes, with relatively small sample sizes (17, 18, 22). One study conducted single-center prospective external validation (13).

### 3.6 Risk of bias and quality assessment

Overall, most prediction models were judged to have a high risk of bias. The results of the PROBAST assessment are presented in Table 3. In the participants domain, eight studies were assessed as having a high risk of bias because they were retrospective studies (10, 12, 14, 17–19, 22, 23). In the predictors domain, the risk of bias was judged as unclear in six studies (19, 24). One study did not report sufficient information regarding the definition and assessment of predictors (24), another did not specify whether predictors were assessed without knowledge of outcome status (19). In the outcome domain, one study was rated as having a high risk of bias (19), while the remaining studies were judged as unclear. This study applied a suboptimal outcome classification method, which may have led to misclassification, and did not report the time interval between predictor assessment and outcome determination. Moreover, none of the included studies reported whether outcome assessment was conducted blinded to predictor information. In the analysis domain, all studies were judged to have a high risk of bias. Three studies had an events-per-variable (EPV) value of less than 10 (18, 20, 22). Concerns regarding applicability were mainly related to participant selection and outcome assessment, reflecting heterogeneity in study populations and delirium diagnostic criteria.

**Table 3:** The risk of bias and applicability of included studies (*n* = 16).

Included studies	Risk of bias				Applicability			Overall	
	Participants	Predictors	Outcome	Analyses	Participants	Predictors	Outcome	Risk of bias	Applicability
Berger et al. (19)	+	?	+	+	−	−	−	+	−
Cai et al. (15)	−	−	?	+	+	−	−	+	+
Cui et al. (16)	−	−	?	+	−	−	−	+	−
Guldolf et al. (10)	+	−	?	+	−	−	−	+	−
Haight and Marsh (22)	+	−	?	+	−	−	−	+	−
Kostalova et al. (20)	−	−	?	+	−	−	−	+	−
Oldenbeuving et al. (13)	−	−	?	+	−	−	−	+	−
Pasinska et al. (23)	+	−	?	+	−	−	−	+	−
Zhou et al. (17)	+	−	?	+	−	−	−	+	−
Wang et al. (24)	−	?	?	+	+	−	−	+	+
Fan et al. (21)	−	−	?	+	+	−	−	+	+
Nakamizo et al. (11)	−	−	?	+	−	−	+	−	−
Klimiec-Moskal et al. (14)	+	−	?	+	−	−	+	−	−
Wischmann et al. (18)	+	−	?	+	−	−	−	+	−
Rex et al. (12)	+	−	?	+	−	−	−	+	−
Kotfis et al. (25)	−	−	?	+	−	−	−	+	−

“+” indicates high risk of bias/low concern regarding application; “−” indicates low risk of bias/high concern regarding applicability; “?” indicates unclear risk of bias/unclear concern regarding applicability.

### 3.7 Meta- analysis of predictors and AUC

**Figure 2:**
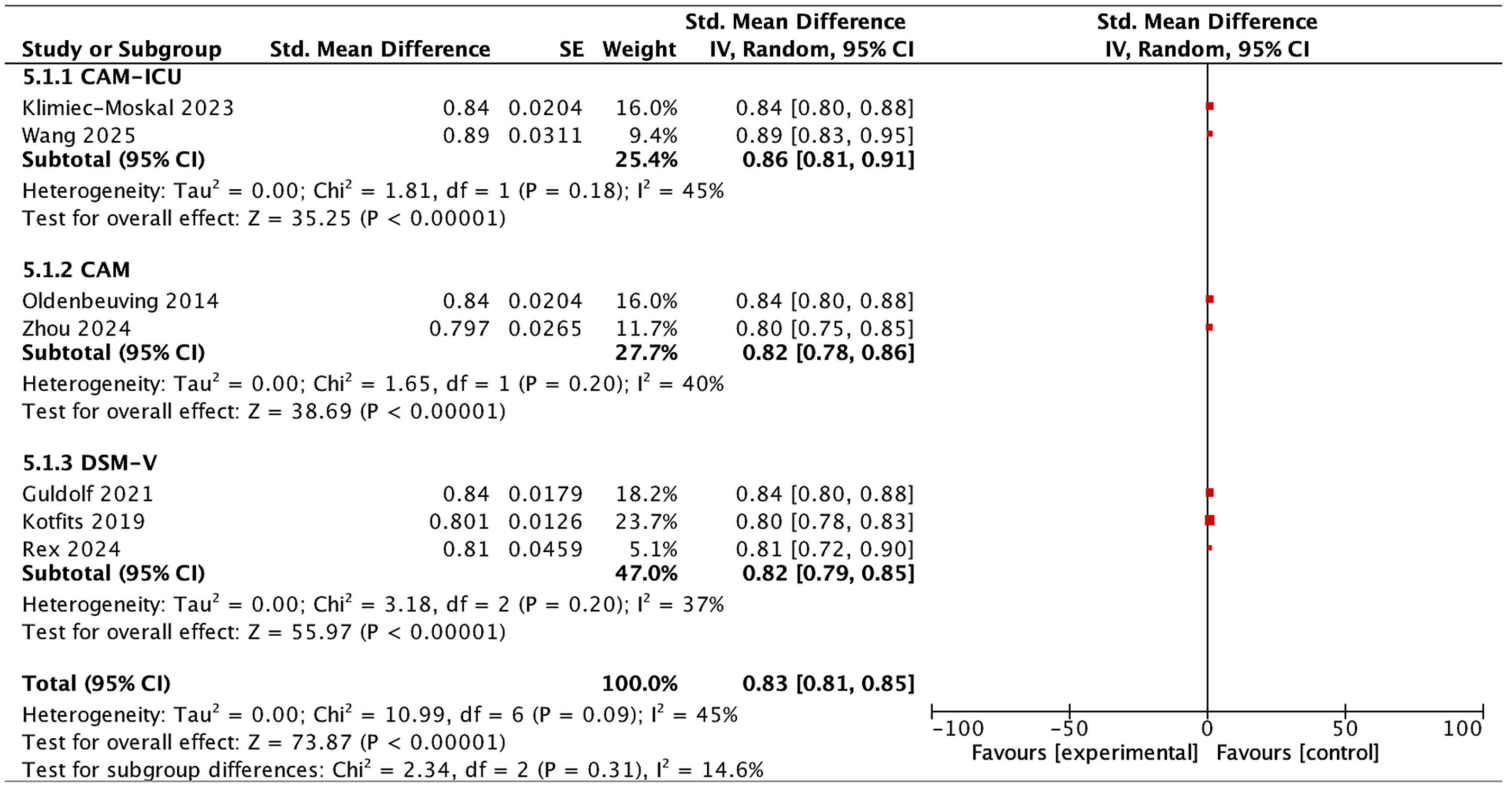
Forest plot of AUC combined effects.

The results of meta-analysis in common predictive factors included age, NIHSS score, NLR, visual impairment and infection (*p* < 0.05) (Table 4). A total of 14 studies reported the AUC values for the prediction models. Among these, four studies provided AUC values without corresponding 95% CI and were therefore excluded from the quantitative synthesis. Of the remaining 10 studies, we excluded one used ICD-10 as diagnostic criteria (19), one used Intensive Care Delirium Screening Checklist as diagnostic criteria (11), and one applied two different delirium assessment tools simultaneously (21). Finally, seven studies were included for the pooled analysis of AUC values (10, 12–14, 17, 24, 25). The pooled AUC was 0.83 (95% CI: 0.81–0.85), with low to moderate heterogeneity (*I*^2^ = 45%), as estimated using a random-effects model (Figure 2). To further investigate potential sources of heterogeneity, subgroup analyses were performed according to the delirium diagnostic criteria applied across studies. Moderate heterogeneity was observed in the CAM-ICU subgroup (*I*^2^ = 45%), DSM-V subgroup (*I*^2^ = 37%), and the CAM subgroup (*I*^2^ = 40%). The pooled AUC was 0.86 (95% CI: 0.81–0.91) for studies using CAM-ICU, 0.82 (95% CI: 0.79–0.85) for studies using DSM-V, and 0.82 (95% CI: 0.78–0.86) for studies using CAM.

**Table 4:** Meta-analysis of common predictive factors.

Predictive factor	Effect model	Pooled effect size				Heterogeneity	
		OR	95% CI	*Z*	*p*	*I*^2^ (%)	*p*
Age (10, 16, 17, 20, 21)	Fixed effect model	1.05	1.03–1.06	6.18	<0.00001	0	0.67
NIHSS (10, 11, 14, 16, 17, 19, 21)	Random effect model	1.28	1.15–1.41	4.69	<0.00001	92	<0.00001
NLR (10, 15, 21, 25)	Fixed effect model	1.11	1.05–1.17	3.85	0.0001	0	0.87
Visual impairment (16, 19, 23)	Random effect model	2.04	1.21–3.44	2.66	0.008	61	0.08
Infection (16, 19)	Fixed effect model	2.19	1.77–2.72	7.24	<0.00001	0	0.32

## 4 Discussion

Among the included studies, the reported incidence of post-stroke delirium varied widely, ranging from approximately 2.7 to 43%. This substantial heterogeneity is likely attributable to the varying patient populations as well as the fundamental differences in delirium assessment methods. Among included studies, five studies used CAM-ICU (14, 16, 21, 22, 24), two employed CAM (13, 17), four employed DSM-V (10, 12, 18, 23), and one relied on ICD-10 diagnostic criteria (19). Compared with previous systematic review, we performed subgroup analyses according to different diagnosis criteria to further explore and reduce potential sources of heterogeneity (26). This lack of standardization complicates both the interpretation of individual model performance and the comparison across studies. Importantly, when prediction models are developed using different outcome definitions, their generalizability to settings employing alternative diagnostic approaches becomes limited. Therefore, the adoption of standardized, validated delirium assessment tools is essential to enhance the generalizability of prediction models and to facilitate robust quantitative comparisons of model performance in future research.

In addition, Wang et al. (24) focused specifically on subarachnoid hemorrhage, with main predictors including alcohol use, coronary heart disease, mechanical ventilation, analgesic use, sedative use, and hypoalbuminemia. Seven studies focused on ischemic stroke, identifying NIHSS score or age as key predictors (10, 11, 14, 17–19, 21). The remaining studies included mixed ischemic and hemorrhagic stroke patients. Mixing different stroke etiologies reduces predictive model accuracy, sensitivity, specificity, and generalization ability by diluting subtype-specific signals, increasing heterogeneity, and raising the risk of overfitting, thus failing to meet clinical prediction needs for different stroke subtypes. Future model development should target specific subtypes rather than a general stroke population.

Age, NIHSS score, NLR, visual impairment, and infection emerged as commonly identified predictors in PSD risk prediction models. The pooled results for the NIHSS scores showed substantial heterogeneity (*I*^2^ = 92%), which may be attributed to differences in statistical handling: five studies handled the NIHSS score as a categorical variable (10, 11, 16, 17, 19), while two studies analyzed it as a continuous variable (14, 21). Future studies should adopt standardized approaches for the measurement and data processing of core predictors to ensure comparability and enable more robust meta-analyses.

Increasing age is associated with neurodegenerative changes in the nervous system, including reduced capacity for neurotransmitter synthesis and cortical neurotransmitter imbalance, which may increase vulnerability to delirium in older adults (27). NIHSS is a well-established tool for quantifying the severity of neurological deficits in patients with stroke, with higher NIHSS scores indicating more extensive and severe brain tissue damage (28). When brain injury involves regions associated with cognition and arousal regulation, such as the frontal lobe, temporal lobe, and thalamus, it may directly disrupt information integration within cortico–subcortical networks, leading to disturbances in the sleep–wake cycle, impaired attention, and memory decline, thereby providing a pathological basis for the development of delirium (29). The NLR is a readily available marker reflecting the magnitude of the systemic inflammatory response. Brain tissue injury can activate the “peripheral–central inflammatory axis,” resulting in an elevated NLR. Central neuroinflammation may directly impair neuronal synaptic function and disrupt the synthesis and transmission of neurotransmitters, such as acetylcholine and *γ*-aminobutyric acid, leading to cognitive dysfunction and ultimately precipitating delirium (10, 25). Furthermore, another studies has shown that adding CRP to clinical models can moderately improve the discrimination and reclassification of stroke patients with delirium (14). CRP may be considered a biomarker for post-stroke delirium in future research. Visual impairment is a common sensory deficit in patients with stroke, including reduced visual acuity, visual field defects, and diplopia. Its association with delirium is primarily attributed to the pathophysiological mechanism of sensory deprivation leading to cognitive disorganization (30, 31). Infection represents one of the most common and potentially modifiable risk factors for delirium in patients with stroke, with pneumonia, urinary tract infection, and intracranial infection being the most frequently observed. Infection-related fever may directly affect cortical arousal, leading to delirium-like manifestations, such as alternating somnolence and agitation (32).

The above five indicators can all be collected and quantified within a short time after patient admission, thereby meeting the clinical need for early and rapid prediction of PSD. Age is a demographic variable readily obtained during the initial admission interview; the NIHSS is a routine bedside assessment scale in stroke care; the NLR is derived from standard blood tests performed on admission; visual impairment can be rapidly identified through brief bedside assessment in conjunction with medical history; and infection is routinely screened for during the acute phase of stroke. Moreover, except for age, which serves as a non-modifiable risk stratification marker, the remaining four factors represent clinically targetable and potentially modifiable intervention points.

The PROBAST assessment indicated that most included models were at high risk of bias, particularly in the participant and analysis domain. Common limitations included lack of external validation, predictor selection based on univariable analysis, inadequate handling of missing data, and a low EPV ratio <10. These issues increase the risk of overfitting. As a result, models may show optimistic performance in derivation datasets but perform poorly in new populations (33). This discrepancy may cause misclassification of patient risk. In addition, improper handling of missing data may introduce selection bias and inaccurate risk estimation (34). These issues not only compromise patient safety but may also increase healthcare burden and reduce the efficiency of resource allocation. Recommended approaches include shrinkage techniques, multiple imputation, and robust internal validation such as bootstrapping or cross-validation. External validation was also often lacking, which limits generalizability. In addition, this study only included studies published in Chinese and English, which may introduce potential selection bias. Overall, given the high risk of bias, the current clinical utility of these models remains limited and should be interpreted with caution (35).

## 5 Conclusion

In conclusion, the ROB assessment indicated a high risk of bias across all included studies, which may have led to overestimation of model performance. Although the pooled AUC of 0.84 suggests moderate to good discrimination, its performance in individual clinical settings may vary markedly, which limits its reliability and clinical applicability. Future research should develop prediction models in accordance with the PROBAST framework to improve methodological rigor, perform multicenter external validation with large sample sizes, and assess model effectiveness and feasibility in real-world clinical practice. These efforts are essential to support the early identification of patients at high risk of post-stroke delirium and to provide reliable tools for clinical decision-making.

## Data Availability

The original contributions presented in the study are included in the article/Supplementary material, further inquiries can be directed to the corresponding authors.
