# Supplementary material for: Prediction models for post-stroke delirium: a systematic review with an exploratory meta-analysis of predictors
Source: Front Neurol. 2026 Jun 18;17:1800718. doi: 10.3389/fneur.2026.1800718 (PMC13322930; doi:10.3389/fneur.2026.1800718)
Supplement: Supplementary file 1 [file Table_1.DOCX]

Supplementary Table S1

| Database | Search strategy | Number  (26.4) |
| --- | --- | --- |
| PubMed | #1 ("stroke"[MeSH Terms] OR "stroke"[tiab] OR "cerebrovascular accident"[tiab] OR "cerebral infarction"[tiab] OR "brain infarction"[tiab] OR "ischemic stroke"[tiab] OR "hemorrhagic stroke"[tiab] OR "cerebral hemorrhage"[MeSH] OR "intracranial hemorrhage"[tiab])  #2 ("delirium"[MeSH Terms] OR "delirium"[tiab] OR "acute confusional state"[tiab] OR "encephalopathy"[tiab] OR "altered mental status"[tiab])  #3 ("risk prediction model"[tiab] OR "predictive model"[tiab] OR "prognostic model"[tiab] OR "risk score"[tiab]] OR "nomogram"[tiab] OR "risk stratification"[tiab] OR "ROC curve"[MeSH])  (#1) AND (#2) AND (#3) | 61 |
| Embase | ((('stroke':ti,ab,kw OR 'cerebrovascular accident':ti,ab,kw OR 'cerebral infarction':ti,ab,kw OR 'brain infarction':ti,ab,kw OR 'ischemic stroke':ti,ab,kw OR 'hemorrhagic stroke':ti,ab,kw OR 'intracranial hemorrhage':ti,ab,kw) OR (('cerebrovascular accident'/exp) OR ('brain hemorrhage'/exp))) AND ((((('delirium'/exp) OR ('delirium':ti,ab,kw)) OR ('acute confusional state':ti,ab,kw)) OR ('encephalopathy':ti,ab,kw)) OR ('altered mental status':ti,ab,kw))) AND ((((((('risk prediction model':ti,ab,kw) OR ('predictive model':ti,ab,kw)) OR ('prognostic model':ti,ab,kw)) OR ('risk score':ti,ab,kw)) OR ('nomogram':ti,ab,kw)) OR ('risk stratification':ti,ab,kw)) OR ('roc curve':ti,ab,kw)) | 260 |
| Web of science | #1 "stroke" OR "cerebrovascular accident" OR "cerebral infarction" OR "brain infarction" OR "ischemic stroke" OR "hemorrhagic stroke" OR "cerebral hemorrhage" OR "intracranial hemorrhage"  #2 "delirium" OR "acute confusional state" OR "encephalopathy" OR "altered mental status"  #3 "risk prediction model" OR "predictive model" OR "prognostic model" OR "risk score" OR "nomogram" OR "risk stratification" OR "ROC curve"  (#1) AND (#2) AND (#3) | 147 |
| Cochrane Library | #1 "stroke" OR "cerebrovascular accident" OR "cerebral infarction" OR "brain infarction" OR "ischemic stroke" OR "hemorrhagic stroke" OR "cerebral hemorrhage" OR "intracranial hemorrhage"  #2 "delirium" OR "acute confusional state" OR "encephalopathy" OR "altered mental status"  #3 "risk prediction model" OR "predictive model" OR "prognostic model" OR "risk score" OR "nomogram" OR "risk stratification" OR "ROC curve"  (#1) AND (#2) AND (#3) | 32 |
| CINAHL | #1 "stroke" OR "cerebrovascular accident" OR "cerebral infarction" OR "brain infarction" OR "ischemic stroke" OR "hemorrhagic stroke" OR "cerebral hemorrhage" OR "intracranial hemorrhage"  #2 "delirium" OR "acute confusional state" OR "encephalopathy" OR "altered mental status"  #3 "risk prediction model" OR "predictive model" OR "prognostic model" OR "risk score" OR "nomogram" OR "risk stratification" OR "ROC curve"  (#1) AND (#2) AND (#3) | 38 |
| CNKI | #1 SU= （脑卒中 + 中风 + 脑梗死 + 脑梗塞 + 脑出血 + 蛛网膜下腔出血 + 脑血管意外 + 脑血管病 + 中风后）  #2 SU= （谵妄 + 急性谵妄 + 意识模糊 + 意识障碍 + 急性意识障碍 + 精神错乱）  #3 SU= （风险预测 + 预测模型 + 预后模型 + 风险评分 + 列线图 + 诺莫图 + ROC曲线 + 模型构建）  (#1) AND (#2) AND (#3) | 110 |
| Wangfang Database | #1 主题 脑卒中 OR 中风 OR 脑梗死 OR 脑梗塞 OR 脑出血 OR 蛛网膜下腔出血 OR 脑血管意外 OR 脑血管病 OR 中风后  #2 主题 谵妄 OR 急性谵妄 OR 意识模糊 OR 意识障碍 OR 急性意识障碍 OR 精神错乱  #3 主题 风险预测 OR 预测模型 OR 预后模型 OR 风险评分 OR 列线图 OR 诺莫图 OR ROC曲线 OR 模型构建  (#1) AND (#2) AND (#3) | 446 |
| CBM | #1 主题 脑卒中 OR 中风 OR 脑梗死 OR 脑梗塞 OR 脑出血 OR 蛛网膜下腔出血 OR 脑血管意外 OR 脑血管病 OR 中风后  #2 主题 谵妄 OR 急性谵妄 OR 意识模糊 OR 意识障碍 OR 急性意识障碍 OR 精神错乱  #3 主题 风险预测 OR 预测模型 OR 预后模型 OR 风险评分 OR 列线图 OR 诺莫图 OR ROC曲线 OR 模型构建  (#1) AND (#2) AND (#3) | 170 |
| Chinese Medical Journal Full-text Database | #1 主题 脑卒中 中风 脑梗死 脑梗塞 脑出血 蛛网膜下腔出血 脑血管意外 脑血管病 中风后  #2 主题 谵妄 急性谵妄 意识模糊 意识障碍 急性意识障碍 精神错乱  #3 主题 风险预测 预测模型 预后模型 风险评分 列线图 诺莫图 ROC曲线 模型构建  (#1) AND (#2) AND (#3) | 15 |
